# A case of anti‐laminin gamma‐1 (p200) pemphigoid with bullous lesions on the lips, hands and feet showing antibodies to desmoglein 1

**DOI:** 10.1002/ski2.101

**Published:** 2022-02-11

**Authors:** F. Kaneko, M. Ozasa, A. Togashi

**Affiliations:** ^1^ Dermatology Unit Southern TOHOKU General Hospital Southern TOHOKU Research Institute for Neuroscience Koriyama Japan

## Abstract

An 87‐years‐old‐man presented with bullous and erosive lesions exclusively on the lips, hands and feet. Histopathological and direct immunofluorescence studies suggested the diagnosis of bullous pemphigoid, but the serum anti‐BP180 antibodies were negative. Strangely, anti‐desmoglein 1 antibodies were positive, although no pemphigus‐like features were found. Indirect immunofluorescence showed IgG reactivity with dermal side of 1 M NaCl‐split skin. Immunoblotting showed positive reactivity with the 200 kDa laminin gamma‐1 but not with type VII collagen. After the administration of prednisolone 20 mg/day, bullous lesions cleared within 2 weeks.

## Graphical abstract

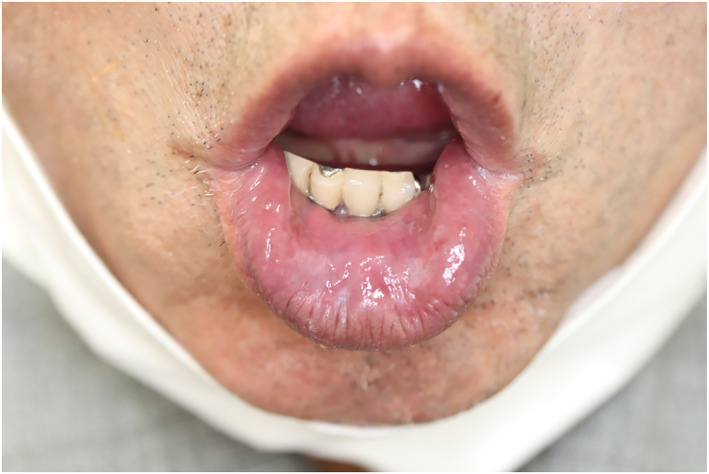

## 1 REPORT OF CASE

**FIGURE 1:**
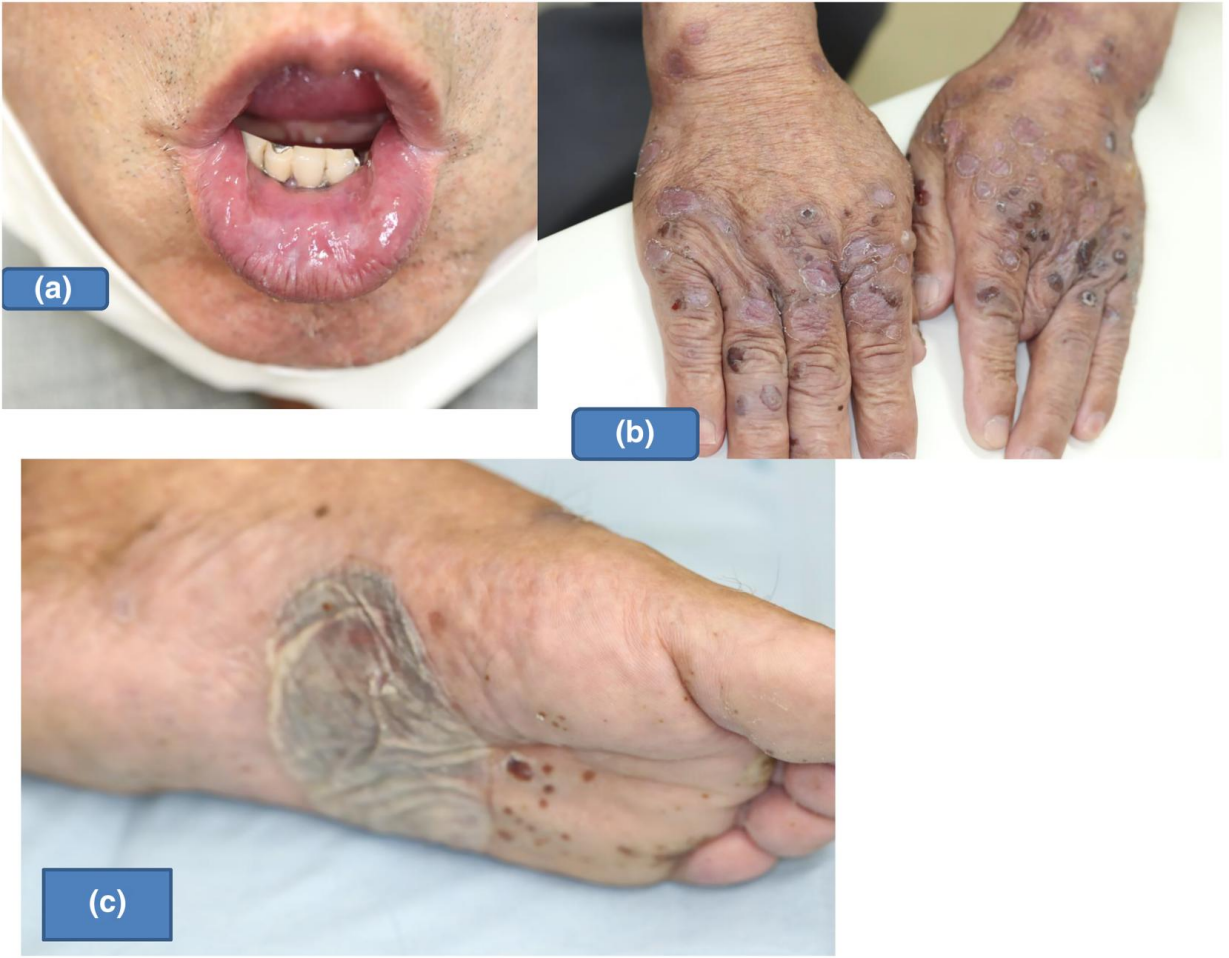
Clinical features (a) on the lips, (b) on the dorsal hands, and (c) soles

An 87‐years‐ole man presented in April 2021, complaining of bullous and erosive lesion exclusively on the lips, dorsa of hands and soles (Figure 1a–c), which had developed over the past 4 weeks. No oral mucosal lesions were observed. Laboratory examinations showed elevated serum IgE (860 IU) and slightly elevated HbA1c level (6.4%), although he was not being treated for diabetes mellitus. Eosinophilia was not found. Chemiluminescent enzyme immunoassays (CLEIAs) showed positive anti‐desmoglein 1 (Dsg1) antibodies (36.2 U/ml: normal <20), although antibodies to Dsg3 and BP180 were negative. ELISA for type VII collagen was negative.

**FIGURE 2:**
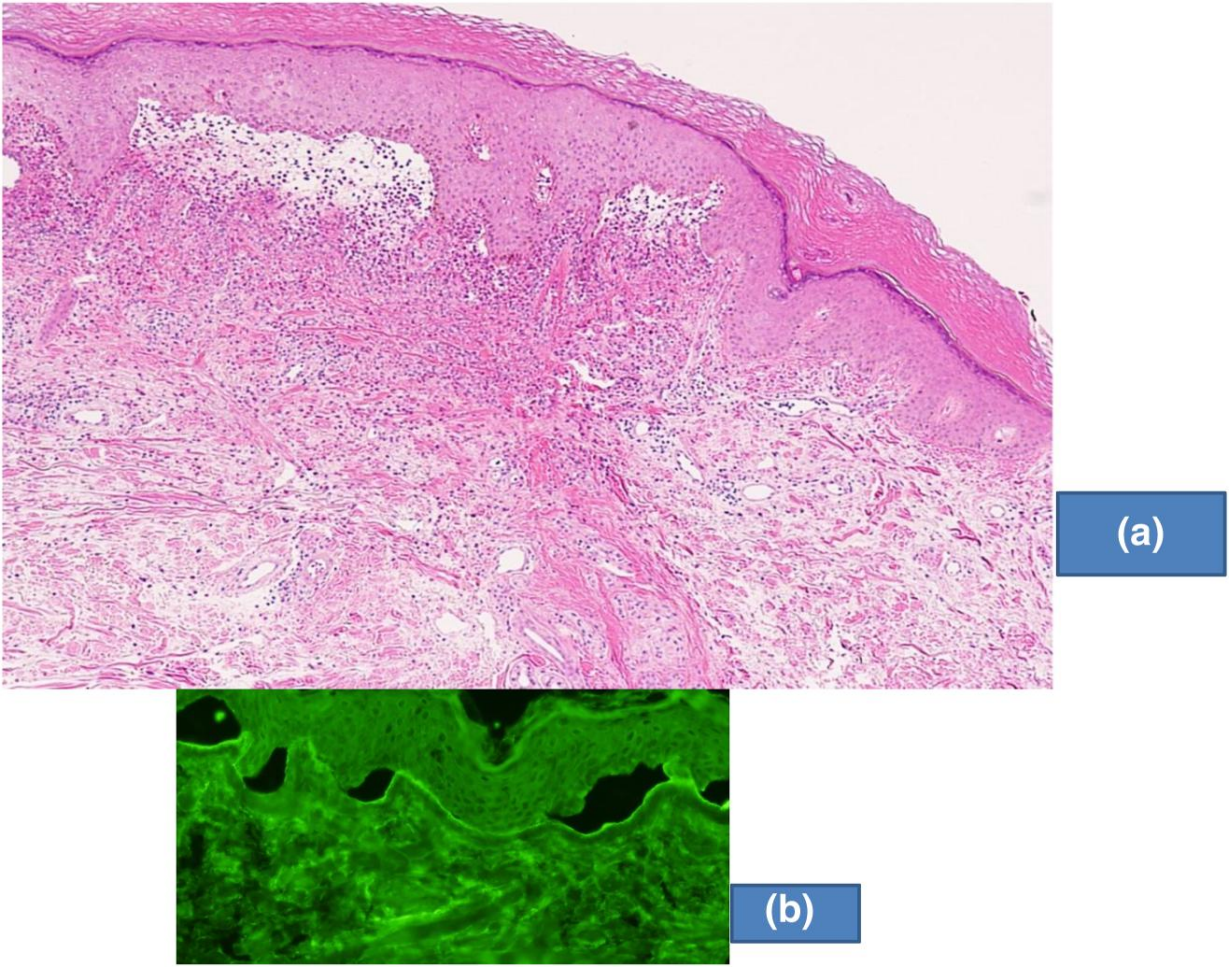
The results of (a) histopathology and (b) indirect immunofluorescence using 1 M NaCl‐split skin at titre 1:40

Bullous pemphigoid (BP) was suspected^1^ and biopsy was taken from the dorsum of the right hand. Histopathology showed subepidermal blister formation with infiltration of eosinophils, neutrophils and lymphocytes (Figure 2a). Direct immunofluorescence showed linear deposits of IgG and C3 at the basement membrane zone (BMZ).

**FIGURE 3:**
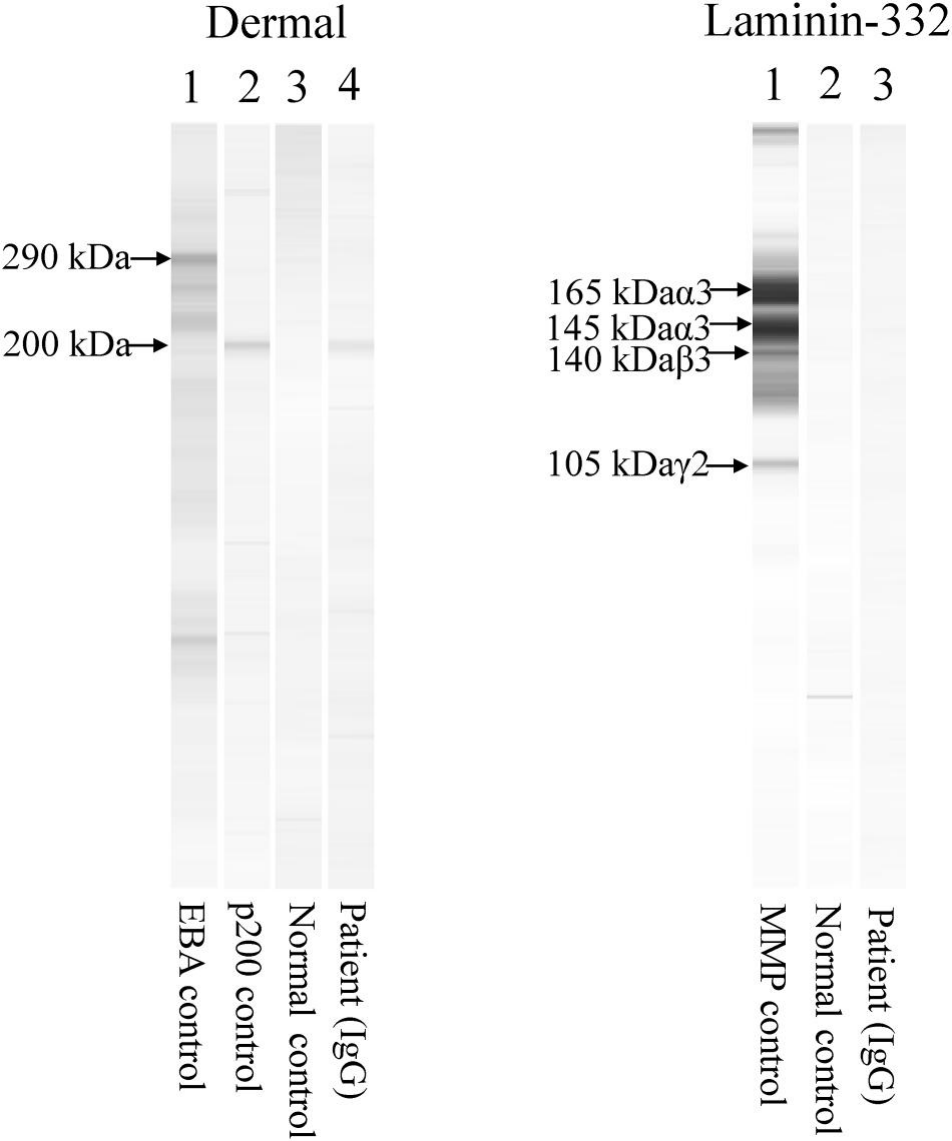
Immunoblotting analysis of the patient's serum. In immunoblotting of normal human dermal extract (Dermal), the patient serum reacted with the 200 kDa laminin gamma‐1, but not with the 290 kDa type VII collagen (left). No positive reactivity was seen in immunoblotting using purified human laminin‐332 (light). (Performed by the Study Group of Kurume University)

We asked the Department of Dermatology, Kurume University School of Medicine (Professor Takekuni Nakama and former professor Takashi Hashimoto) to perform various immuno‐serological tests. Indirect immunofluorescence using 1 M NaCl‐split skin showed IgG reactivity with dermal side of the split (Figure 2b). Immunoblotting of normal human dermal extract were positive to the 200 kDa laminin gamma‐1 but negative to type VII collagen. No reactivity to laminin −332 was seen (Figure 3).

The diagnosis of anti‐laminin gamma‐1 (p200) pemphigoid was made, and oral prednisolone 20 mg/day started, leading to clearance of all the lesions after 2 weeks without scars and post‐inflammatory pigmentation. The prednisolone dose was tapered to 5 mg/day without relapse. However, anti‐Dsg1 antibodies continued to be positive.

## 2 DISCUSSION

For the precise diagnosis of distinct autoimmune bullous diseases, various immune‐serological examinations, including CLEIA for Dsg1, Dsg3, BP180, are performed, in addition to clinical and histopathological examinations and direct immunofluorescence.

Anti‐laminin gamma‐1 (p200) pemphigoid is a relatively newly identified autoimmune subepidermal bullous disease with IgG autoantibodies to the 200 kDa antigen present in the lamina lucida, which was identified as laminin gamma‐1.^2^, ^3^, ^4^ This case was designated as anti‐p200 pemphigoid, and the name anti‐laminin gamma‐1 pemphigoid was proposed after the autoantigen was identified.

Our patient exhibited BP‐like bullous lesions only on the dorsal surfaces of the hands and soles, as well as erosive lesions on the lips. Histopathological features and direct immunofluorescence results were compatible with BP, although BP180 CLEIA was negative. Finally, from the results of indirect immunofluorescence using split‐skin and various immunoblotting analyses, a final diagnosis of anti‐laminin gamma‐1 pemphigoid was confirmed.

According to the most recent report of the detailed analysis of 68 anti‐laminin gamma‐1 pemphigoid patients by Kridin and Ahmed,^5^ BP‐like skin lesions associated with urticarial plaques were found on the entire body including head, face and palmoplantar regions. However, cases only with skin lesions on the dorsal hands and feet was not reported. Therefore, the distribution of the skin lesions seen in our patient is very rare in this disease.

Mucosal involvement was found in 38.5% of cases and scars/milia developed in 15.7% of cases.^5^ There is also another report of the anti‐laminin gamma‐1 pemphigoid cases involving the mucous membranes in this disease.^6^ Thus, the erosive lesion on the lips was considered to be a symptom of anti‐laminin gamma‐1 pemphigoid patients, although our case did not leave either scarring or milia.

Anti‐Dsg1 antibodies were continuously positive in our case. However, because no pemphigus‐like lesions were found, the pathogenic activity of the anti‐Dsg1 antibodies is unclear and may be secondary non‐pathogenic antibodies. Although disappearing bullous and erosion lesions from the patient by taking 5 mg of predonisolone, he was attacked by pneumonia which should be admitted and treated with antibiotics. During the treatment for pneumonia he was suddenly developing to senile dementia in two of 3 month later. So, we could not take the permission for publication of the clinical features from himself.

## CONFLICT OF INTEREST

None to declare.

## Data Availability

Data supporting the findings of this study are available from the corresponding author upon reasonable request.
